# 

**Published:** 1876-11

**Authors:** 


### TABLE OF CONTENTS.

SELECTIONS—

Complementary Parts of Disease. By Alex McBride, M.D., Berea, Ohio...... 641
Treatment of Cholera Infantum. By Edward Waldo Emmerson, M.D........ 650
Injecting the Male Bladder Without the Aid of a Catheter, and some of its Advantages........................................................ 654
Cupping and Ice in Acute Gastritis. By S. D. Chase, M.D., of Osage, Iowa.. 657
Itinerant Pile-Surgeons and their Secret. By Edmund Andrews, A.M., M.D.. 659
Stricture of the Urethra. Clinic, by B. F. Maury, M.D., Phila............... 662
Scarlet Fever. By Lunsford P. Yandell, Jr., M.D., Louisville................ 665
On the External Uses of Hydrate of Chloral. By Dr. Wm Craig, F. R. S. E.. 667

EXTRACTS AND GLEANINGS—

Local Use of Bromide of Potassium—671. Hydrate of Chloral to Prevent a Recurrence of Chill and Fever—673. The Management of Diphtheritic Paralysis—673. Liquor Potassæ in Chlorosis—675. Treatment of Sprain—676. Treatment of Remittent Fever—677. A Useful Mode of Administering Chloroform—678. Cholera Infantum—679. The Menses in Phthisical Patients—681. The Abortive Treatment of Whooping Cough by Sulphate of Quinine—681. The Prevention of Masturbation—682. The Diet for Gout—682. Management of Diphtheria—683. Hypodermic Injections in Glandular Enlargement—684. A Multiple Antidote—685. Sulphurous Acid in Enteric Fever—686. Esmarch's Bandage for Chronic Ulcers—686. Kava-Kava, a Remedy for Gonorrhœa—686. Nasal Catarrh Treated Locally—687. Priapism—687. Gout, Erythema Nodosum—687. Removal of Foreign Bodies from the Ear—687. Cholera Infantum—688. Incompatibility of Iodide and Chlorate of Potassium—688. Sulphate of Cinchonidia—688. Acute Rheumatism—689. Sea Water in the Treatment of Obesity—689. Syrupus Cinchonæ Alcoholicus—689. Treatment of Pruritus Vulvæ—690. The "Bran Bread" Question—690. Coal Oil—691. Belladonna in Asthma—691. Carbolic Acid in Treatment of Whooping Cough—682. Oil of Sandal-Wood in the Treatment of Gonorrhœa—693. Treatment of Diabetes Mellitus by Glycerin—693. Sulpho-Carbolate of Soda in Fever—694. Fatal Result of Injection of Chloral—694 Glycerole of Subacetate of Lead—695. Pimples on the Face—696. Hydro-Chloral by the Rectum in the Vomiting of Pregnancy—696. Muriate of Ammonia in Enlargement of the Spleen—696. A Sensible precaution—696. On the Methods for Detecting Albumen in Urine—697. Mesmeric Anæsthesia—697. "Universal Disinfecting Powder"—698. Milk as a Vehicle for Bromide of Potassium—698. Van Swienten's Liquor—698. Alum, Tannin, and Oxide of Zinc in Sticks—699. Powder for Producing Ozone—699. Extract of Beef—699. Camphorated Ether in Erysipelas—699. Loomis' Tonic—699.

EDITORIAL AND MISCELLANEOUS—

To Delinquents........................................................ 700
Liver Medicine........................................................ 700
For the Cure of Uterine Diseases.................................... 701
Personal and Book Notices............................................ 703

